# Emotional intelligence and clinical decision-making confidence in nurses: the chain mediating effect of creative self-efficacy and self-directed learning

**DOI:** 10.3389/fmed.2025.1708634

**Published:** 2025-12-10

**Authors:** Lin-xia Tang, Wen-yu Yue, Xiao-qin Ma

**Affiliations:** School of Nursing, Zhejiang Chinese Medical University, Hangzhou, China

**Keywords:** clinical nurse, emotional intelligence, creative self-efficacy, self-directed learning, clinical decision-making confidence, chain mediating effect

## Abstract

**Background:**

Clinical decision-making competence underpins autonomous nursing practice. While external influences are documented, context-dependent psychological mechanisms linking emotional intelligence (EI) to clinical decision-making confidence (CDMC) remain unexplored. Using social cognitive theory, this cross-sectional study of 1,126 nurses from 12 Zhejiang tertiary hospitals examined EI’ s influence on CDMC via sequential mediation by creative self-efficacy (CSE) and self-directed learning (SDL), moderated by department stress and clinical experience.

**Methods:**

Nurses completed measures of EI (WLEIS), CDMC (CDMSCS), CSE, and SDL. Analyses used structural equation modeling (SEM) with bias-corrected bootstrapping and multi-group SEM with Bonferroni correction to test moderation by department type (high-stress: ICU/emergency/OR vs. non-high-stress) and experience (≤5 vs. >5 years).

**Results:**

The model showed excellent fit (*χ^2^/df* = 2.999, *RMSEA* = 0.030, *CFI* = 0.963, *GFI* = 0.934). EI predicted CDMC directly (27.16% of total effect) and indirectly via CSE (23.16%), SDL (25.26%), and sequential mediation (24.42%; 72.84% total indirect effect). Moderation revealed: In high-stress units (n = 159), the EI → CSE → CDMC path was stronger (*β* = 0.28, 95% CI [0.22, 0.35] vs. non-high-stress *β* = 0.21, 95% CI [0.17, 0.26]; Δ*β* = 0.07, *p* = 0.012). Conversely, EI → SDL → CDMC dominated in non-high-stress units (*β* = 0.27 vs. 0.20; Δ*β* = 0.07, *p* = 0.018), particularly pediatrics/gynecology. For nurses with ≤5 years’ experience (n = 254), sequential mediation (EI → CSE → SDL → CDMC; *β* = 0.31, 95% CI [0.25, 0.38]) was primary, with negligible direct effects (*β* = 0.08, *p* = 0.12). Nurses with >5 years’ experience (n = 872) showed strengthened direct EI → CDMC effects (*β* = 0.19, *p* = 0.003) and reduced SDL reliance (Δ*β* = 0.12, *p* = 0.007), suggesting experience compensates for psychological resource utilization.

**Conclusion:**

EI enhances CDMC through context-contingent pathways: CSE is pivotal in high-stress environments, SDL prevails in non-high-stress units (notably pediatrics/gynecology), and experiential knowledge supersedes mediation pathways with seniority. These findings challenge uniform decision-making models, urging healthcare systems to implement context/experience-specific strategies—such as stress-adapted EI training for critical care nurses and experiential integration for seniors—to optimize clinical decision-making across nursing settings.

## 1 Background

Nursing practice has evolved from physician-directed care toward autonomous clinical decision-making. In this paradigm, decision-making confidence—a nurse’ s subjective belief in their capacity to make sound judgments—serves as a cornerstone of professional identity. This confidence directly impacts patient safety; low confidence correlates with delayed interventions and a 23% increase in preventable adverse events in acute care settings (1, 2). As a dynamic cognitive process, clinical decision-making confidence integrates evidence-based judgment, critical reflection, and contextual adaptation. It operates within high-stakes environments where errors can carry life-threatening consequences (3–5).

Contemporary research identifies emotional intelligence (EI)—the capacity to perceive, regulate, and utilize emotions (6)—as a predictor of decision-making confidence. However, studies predominantly examine external factors [e.g., education, experience (7–10)], neglecting the psychological mechanisms through which EI operates. In time-pressured clinical scenarios (e.g., ICU emergencies), intensive care unit studies show that emotional dysregulation impairs working memory capacity by approximately 30%. This subsequently reduces diagnostic accuracy by 31% in complex clinical cases (11). Social cognitive theory (SCT) posits that EI enhances self-regulatory resources (12). These resources first bolster creative self-efficacy (CSE), defined as the belief in one’s ability to generate innovative solutions to clinical problems (13). Nurses with higher CSE are more likely to engage in self-directed learning (SDL) (14), as their confidence in innovative problem-solving motivates them to seek learning opportunities that refine clinical judgments (15, 16). Empirical evidence supports EI’s effect on CSE (*β* = 0.42, *p* < 0.05) (17) and the CSE–SDL link (*β* = 0.61, *p* < 0.001) (16). Yet, the sequential pathway EI → CSE → SDL → decision-making confidence remains untested in nursing contexts.

**Figure 1:**
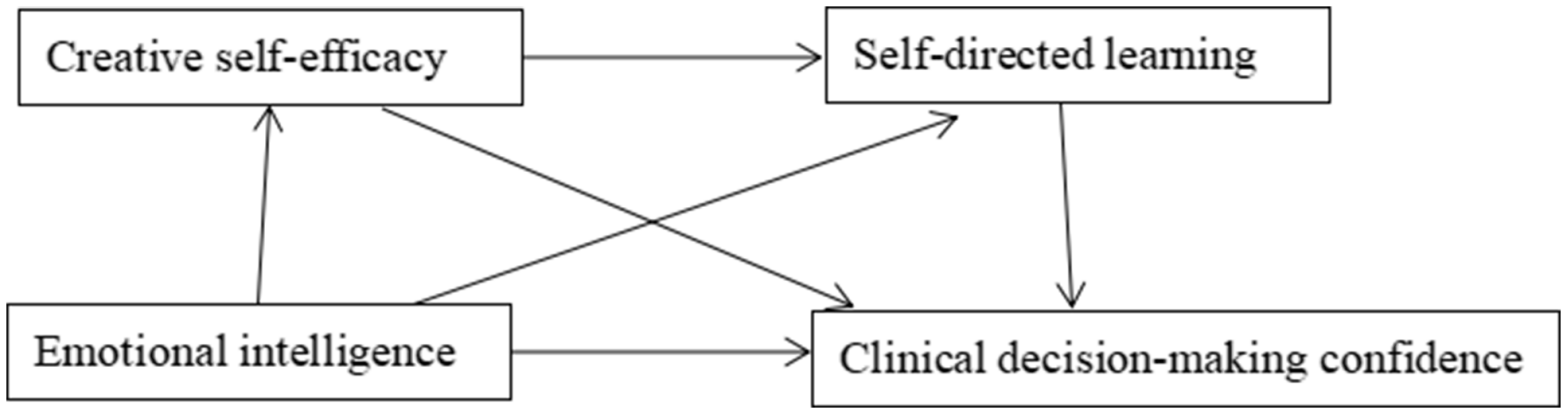
Conception model.

Current sequential mediation models, often adapted from educational psychology (18), fail to account for nursing-specific constraints: (1) life-critical decision consequences (vs. academic/business contexts), (2) interdisciplinary power dynamics inhibiting autonomous action (19), and (3) time pressure requiring decisions within seconds. For instance, business models emphasize iterative experimentation (20), whereas nursing demands immediate protocol application—a context where SDL’ s role in translating knowledge to action becomes paramount (15). Thus, this cross-sectional study tests an SCT—based sequential mediation model to elucidate whether CSE and SDL transmit EI’ s effects to decision-making confidence (Figure 1), providing a foundation for contextually targeted interventions.

The study advances four hypotheses:

*H1:* Emotional intelligence positively influences clinical decision-making confidence.

*H2:* Creative self-efficacy mediates the relationship between emotional intelligence and clinical decision-making confidence.

*H3:* Self-directed learning mediates the relationship between emotional intelligence and clinical decision-making confidence.

*H4:* Creative self-efficacy and self-directed learning sequentially mediate the relationship between emotional intelligence and clinical decision-making confidence (EI → CSE → SDL → CDC).

## 2 Methods

### 2.1 Participants

The minimum required sample size was determined using Kline’ s guideline of 10–20 participants per questionnaire item (21). The study instruments comprised 102 items total (emotional intelligence: 16 items; creative self-efficacy: 14 items; clinical decision-making confidence: 12 items; self-directed learning: 60 items). Thus, the minimum required sample size ranged from 1,020 to 2,040 participants. Accounting for a 15% attrition rate, the final target sample size was 1,173 to 2,346 participants.

### 2.2 Measurements

This study utilized four validated questionnaires to measure the key variables: emotional intelligence, creative self-efficacy, self-directed learning, and clinical decision-making confidence. All instruments were validated scales with established psychometric properties.

#### 2.2.1 General Information Questionnaire

A researcher-designed questionnaire was used to collect participants’ background information across four dimensions: (1) sociodemographic factors (age, gender, marital status, per capita monthly household income), (2) academic background (educational background), (3) institutional factors (hospital type, employment method, department), and (4) professional experience factors (academic title, research project participation experience, article publication experience, academic conference participation experience, years of service).

#### 2.2.2 Wong and Law Emotional Intelligence Scale

Developed by Wong and Law, the WLEIS assesses emotional intelligence across four dimensions: self-emotion appraisal (4 items), others’ emotion appraisal (4 items), use of emotion (4 items), and regulation of emotion (4 items), totaling 16 items (22). Responses are rated on a 7-point Likert scale (1 = strongly disagree to 7 = strongly agree). The Chinese adaptation (Wang, 2023) demonstrated acceptable reliability (total Cronbach’ s *α* = 0.837; subscales: self-appraisal α = 0.773, others’ appraisal α = 0.542, use of emotion α = 0.796, regulation α = 0.844) (23). Evidence of validity includes established factorial structure and cross-cultural applicability, though confirmatory fit indices (CFI, RMSEA) were not reported for the Chinese version.

#### 2.2.3 Clinical Decision-Making Self-Confidence Scale

Hart et al. developed the 12-item CDMSCS to measure nurses’ clinical decision-making confidence during acute patient deterioration, with two dimensions: respiratory/cardiac events (8 items) and neurological events (4 items) (4). It uses a 5-point Likert scale (1 = not at all confident to 5 = extremely confident). The Chinese version in this study showed strong reliability (Cronbach’ s *α* = 0.939) and excellent model fit for the two-factor structure: χ^2^/df = 4.93, CFI = 0.972, TLI = 0.945, RMSEA = 0.078, GFI = 0.963, AGFI = 0.903, supporting robust construct validity.

#### 2.2.4 Four-item measure of creative self-efficacy

Tierney and Farmer developed this unidimensional 4-item scale to assess confidence in generating creative solutions in work contexts (13). Responses use a 7-point Likert scale (1 = strongly disagree to 7 = strongly agree). The Chinese version demonstrated high internal consistency (Cronbach’ s *α* = 0.924) and strong validity evidence via confirmatory factor analysis: χ^2^/df = 2.812, CFI = 0.951, TLI = 0.911, RMSEA = 0.063, GFI = 0.937, AGFI = 0.876, confirming its unidimensional structure and cross-cultural applicability.

#### 2.2.5 Self-rating scale of self-directed learning

Williamson developed the SRSSDL as a 60-item instrument measuring self-directed learning across five dimensions (e.g., planning, implementation, evaluation) (24). It employs a 5-point Likert scale (1 = never to 5 = always). The Chinese adaptation evidenced excellent reliability (Cronbach’ s *α* = 0.966; retest reliability = 0.855) and validity (Content Validity Index = 0.963) (25). Confirmatory fit indices (CFI, RMSEA) were not reported in the original or adapted versions, as validation relied on exploratory factor analysis and content validity.

### 2.3 Date collation

Data collection utilized Wenjuanxing,^1^ a secure web-based survey platform. The research team implemented multiple quality assurance measures: (1) Mandatory response formatting for all items to prevent missing data; (2) IP restriction protocols to ensure single participation; (3) Temporal validation through embedded timestamp tracking. Participants received standardized instructions detailing study objectives, data confidentiality protocols (anonymous identifiers), and voluntary participation rights. To enhance response rates, compliant participants received ¥10 compensation via We Chat Pay, approved by the institutional ethics committee. The research team maintained daily monitoring throughout the 14-day data collection window, resolving technical queries within 24 h through a dedicated hotline.

### 2.4 Statistical analysis

During data organization, two researchers independently cross-checked participant data for completeness and accuracy. Epidata 3.2 was used for database construction, with dual data entry by separate personnel. SPSS 25.0 performed descriptive statistics [mean ± SD for normal data; median (IQR) for non-normal data] and Pearson correlations. Structural equation modeling (SEM) in AMOS 24.0 evaluated path coefficients and model fit (χ^2^/df < 5.0, RMSEA ≤ 0.08, CFI ≥ 0.90, GFI ≥ 0.90) (26). Bias-corrected bootstrapping (5,000 samples) tested indirect effects with 95% CIs (27). Additionally, to address potential contextual influences, multi-group structural equation modeling (MSEM) was conducted to test the moderating effects of department type [high-stress units (ICU, emergency, operating room) vs. non-high-stress units (e.g., pediatrics, gynecology, internal/external medicine)] and years of service (≤5 years vs. >5 years) on the proposed mediation pathways. The significance of path coefficient differences between groups was determined by Δχ^2^ tests with Bonferroni correction (*α* = 0.05).”

### 2.5 Ethical considerations

The study received approval from the Ethics Committee of Zhejiang Chinese Medical University (20250328–1) and adhered to Declaration of Helsinki principles and STROBE guidelines. Comprehensive protocols protected participant rights, including informed consent, confidentiality measures, and voluntary participation provisions. All participants signed the informed consent form. Data analysis employed anonymized identifiers, prioritizing participant privacy while maintaining research integrity.

## 3 Results

### 3.1 General information of respondents

All 1,126 collected questionnaires were valid (100% response rate). Participants were predominantly female (96.6%), with 41.1% aged 30–40 years, as depicted in Table 1.

**Table 1:** General information of respondents [*n* = 1,126, case (percentage,%)].

Variable		N (%)	M (SD)	*t*/*F*	*P*
Age (y)	20–30	335 (29.8)	45.45 ± 7.48	11.491	0.000**
	30–40	463 (41.1)	47.63 ± 7.27		
	40–50	247 (21.9)	48.57 ± 6.34		
	>50	81 (7.2)	48.62 ± 6.49		
Gender	Male	35 (3.1)	47.23 ± 8.20	−0.026	0.979
	Female	1,091 (96.9)	47.26 ± 7.16		
Marital status	Unmarried	319 (28.3)	45.41 ± 7.51	15.087	0.000**
	Married	779 (69.2)	48.00 ± 6.91		
	Divorce or widowed	28 (2.5)	47.64 ± 7.54		
Educational background	Specialist and below	44 (3.9)	44.20 ± 7.98	4.172	0.016*
	Undergraduate	1,034 (91.8)	47.39 ± 7.11		
	master’s degree or above	48 (4.3)	47.23 ± 7.72		
Per capita monthly household income	≤5,000	94 (8.3)	45.71 ± 9.02	5.988	0.000**
	5,000–8,000	402 (35.7)	46.57 ± 6.59		
	8,000–10,000	254 (22.6)	47.21 ± 7.06		
	≥10,000	376 (33.4)	48.41 ± 7.23		
Academic title	Nurse	107 (9.5)	44.36 ± 8.34	10.684	0.000**
	Primary nurse	418 (37.1)	46.85 ± 7.05		
	Nurse-in-charge	498 (44.2)	47.77 ± 7.02		
	Deputy chief nurse or chief nurse	103 (9.1)	49.49 ± 6.22		
Hospital type	Tertiary hospitals	1,105 (98.1)	47.24 ± 7.14	−0.690	0.490
	Secondary hospital	21 (1.9)	48.33 ± 9.60		
Research project participation experience	No	852 (75.7)	46.81 ± 7.21	−3.738	0.000**
	Yes	274 (24.3)	48.66 ± 6.94		
Article publication experience	No	518 (46.0)	46.23 ± 7.32	−4.469	0.000**
	Yes	608 (54.0)	48.14 ± 6.96		
Academic conference participation experience	No	524 (46.5)	46.40 ± 7.57	−3.719	0.000**
	Yes	602 (53.5)	48.00 ± 6.76		
Employment method	Labor dispatch	233 (20.7)	46.41 ± 7.35	6.347	0.002**
	Contract	153 (13.6)	49.02 ± 7.23		
	Authorized strength	740 (65.7)	47.16 ± 7.08		
Years of service (y)	1–5	254 (22.6)	44.90 ± 7.48	11.539	0.000**
	6–10	202 (17.9)	47.30 ± 7.21		
	10–15	296 (26.3)	47.81 ± 7.50		
	16–20	162 (14.4)	47.38 ± 5.77		
	≥21	212 (18.8)	49.19 ± 6.63		
In the department	Internal medicine	391 (34.7)	47.83 ± 7.12	1.861	0.063
	Surgery	321 (28.5)	47.35 ± 6.83		
	ICU	44 (3.9)	48.55 ± 6.29		
	Emergency room	32 (2.8)	46.56 ± 6.91		
	Operating room	83 (7.4)	46.59 ± 8.15		
	Department of pediatrics	24 (2.1)	45.08 ± 7.09		
	Department of gynecology	22 (2.0)	47.00 ± 5.77		
	Obstetrical department	52 (4.6)	44.52 ± 7.84		
	Other	157 (13.9)	47.08 ± 7.62		

*P < 0.05, **P < 0.01.

### 3.2 Intervariable relationships

Emotional intelligence correlated strongly with creative self-efficacy (*r* = 0.783, *p* < 0.001) and self-directed learning (*r* = 0.637, *p* < 0.001). Creative self-efficacy and self-directed learning were positively associated (*r* = 0.613, *p* < 0.001), and both predicted clinical decision-making confidence (*r* = 0.464 and 0.628, respectively; *p* < 0.001), as depicted in Table 2.

**Table 2:** Analysis results of the correlation (r value) between nurses’ emotional intelligence, clinical decision-making confidence, creative self-efficacy, and self-directed learning.

Items	EI	CDS	SDL	CSE
EI	1.000	–	–	–
CDS	0.491**	1.000	–	–
SDL	0.637**	0.628**	1.000	–
CSE	0.783**	0.464**	0.613**	1.000

**P < 0.001. EI, emotional intelligence; CDS, clinical decision-making; SDL, self directed learning; CSE, creative self-efficacy.

### 3.3 Common method bias assessment

Exploratory factor analysis yielded 19 factors with eigenvalues >1. The primary factor explained 34.71% of variance, below the 40% threshold (28), indicating negligible common method bias.

### 3.4 Mediating effect analysis

**Figure 2:**
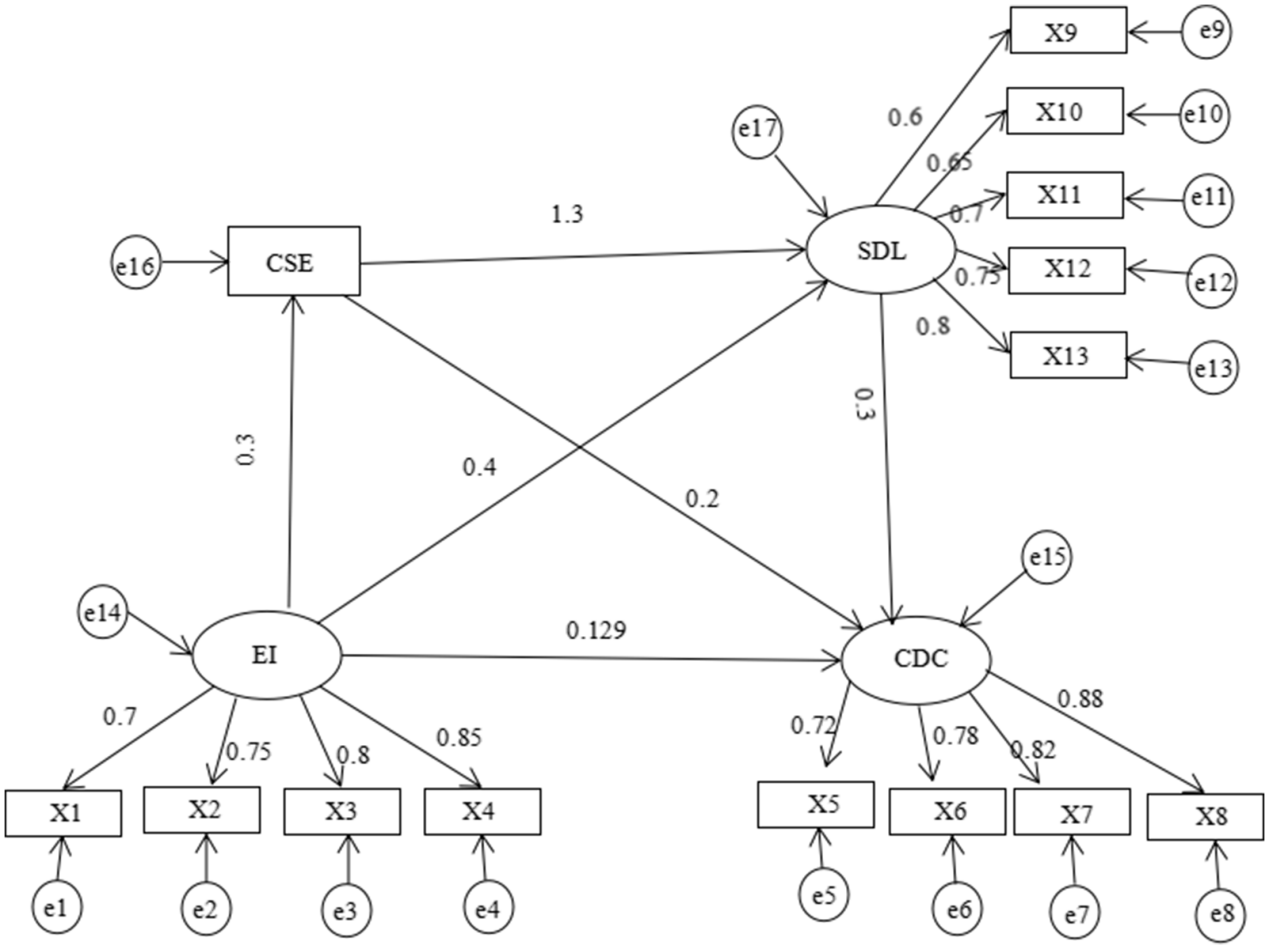
Chain-mediation model of emotional intelligence, creative self-efficacy, self-directed learning, clinical decision-making confidence. X1, self-emotion appraisal; X2, others’ emotion appraisal; X3, use of emotion; X4, regulation of emotion; X5, identification; X6, assessment; X7, intervention; X8, evaluation; X9, learning awareness; X10, learning strategies; X11, learning behavior; X12, learning evaluation; X13, interpersonal relationships.

The structural equation model exhibited excellent fit (*χ^2^/df* = 2.999, *p* < 0.001; *RMSEA* = 0.030; *CFI* = 0.963; *GFI* = 0.934). Figure 2 present the relationship of emotional intelligence, creative self-efficacy, self-directed learning, clinical decision-making confidence.

Bootstrap method with 5,000 times resamples was used to test mediation effects, and the results shown in Table 3 suggested a statistically significant difference in mediation effects. The direct effect between emotional intelligence and clinical decision-making confidence accounts for 27.16% of the total effect, which supports hypothesis 1. The indirect effect between emotional intelligence and clinical decision-making confidence accounted for 72.84% of the total effect, and 95%CI of the three mediating effect paths did not include 0 after Bootstrap analysis, which confirmed that the mediating effect was significant. The indirect effect composed of emotional intelligence → creative self-efficacy → clinical decision-making confidence accounts for 23.16% of the total indirect effect, which supports hypothesis 2; The indirect effect composed of emotional intelligence → self-directed learning → clinical decision-making confidence accounts for 25.26% of the total indirect effect, which supports hypothesis 3; The indirect effect composed of emotional intelligence → creative self-efficacy → self-directed learning → clinical decision-making confidence accounts for 24.42% of the total indirect effect, which supports hypothesis 4.

**Table 3:** Chain mediating effect on emotional intelligence, creative self-efficacy, self-directed learning and clinical decision-making confidence (*n* = 1,126).

Paths	Estimate value (standardized)	S.E.	Bootstrapping 95%CI		Account for the total effect (%)
			Lower	Higher	
Total effect	0.475	0.017	0.406	0.540	100
Direct effect	0.129	0.052	0.027	0.284	27.16
Total indirect effect	0.346	0.014	0.214	0.477	72.84
Ind1	0.110		0.075	0.145	23.16
Ind2	0.120		0.086	0.166	25.26
Ind3	0.116		0.080	0.152	24.42

Ind1, emotional intelligence→creative self-efficacy→clinical decision-making confidence; Ind2, emotional intelligence→self-directed learning→clinical decision-making confidence; Ind3, emotional intelligence→creative self-efficacy→self-directed learning→clinical decision-making confidence.

### 3.5 Moderating effects of contextual factors

Multi-group SEM revealed significant moderation by department type and years of service (Table 4). Department type: In high-stress units (ICU/emergency/operating room, *n* = 159), the indirect effect of emotional intelligence on clinical decision-making confidence through creative self-efficacy was stronger (*β* = 0.28, 95% *CI* [0.22, 0.35]) than in non-high-stress units (*β* = 0.21, 95% *CI* [0.17, 0.26]; Δ*β* = 0.07, *p* = 0.012). Conversely, the sequential mediation path (emotional intelligence → self-directed learning → clinical decision-making confidence) was significantly stronger in non-high-stress units (*β* = 0.27, 95% *CI* [0.22, 0.33] vs. *β* = 0.20, 95% *CI* [0.15, 0.26]; Δ*β* = 0.07, *p* = 0.018), particularly in pediatric/gynecological departments where holistic patient-family communication is prioritized.

**Table 4:** Moderation analysis of department type and years of service.

Pathway	High-stress units (*β*)	Non-high-stress units (*β*)	Δ*β* (*P*)	≤5 years (*β*)	>5 years (*β*)	Δ*β* (*P*)
EI → CSE → CDC	0.28 [0.22, 0.35]	0.21 [0.17, 0.26]	0.07 (0.012*)	0.24 [0.18, 0.30]	0.22 [0.18, 0.27]	0.02 (0.31)
EI → SDL → CDC	0.20 [0.15, 0.26]	0.27 [0.22, 0.33]	−0.07 (0.018*)	0.29 [0.24, 0.35]	0.22 [0.18, 0.27]	0.07 (0.021*)
EI → CSE → SDL → CDC	0.25 [0.19, 0.31]	0.24 [0.19, 0.30]	0.01 (0.63)	0.31 [0.25, 0.38]	0.22 [0.18, 0.27]	0.09 (<0.001**)
EI → CDC (direct)	0.15 [0.09, 0.22]	0.29 [0.24, 0.35]	−0.14 (<0.001**)	0.08 [−0.02, 0.18]	0.19 [0.10, 0.28]	−0.11 (0.003**)

EI, Emotional Intelligence; CSE, Creative Self-Efficacy; SDL, Self-Directed Learning; β, tandardized path coefficient; 95% CI in brackets; **p < 0.01, *p < 0.05.

Years of service: For nurses with ≤5 years of experience (*n* = 254), emotional intelligence’s direct effect on clinical decision-making confidence was negligible (*β* = 0.08, *p* = 0.12), while the sequential mediation effect (emotional intelligence → creative self-efficacy → self-directed learning → confidence) dominated (*β* = 0.31, 95% *CI* [0.25, 0.38]). In contrast, nurses with >5 years of service (*n* = 872) showed a strengthened direct effect (*β* = 0.19, *p* = 0.003) and reduced reliance on self-directed learning pathways (Δ*β* = 0.12, *p* = 0.007), suggesting experiential knowledge compensates for psychological resource utilization.

## 4 Discussion

This study advances understanding of the psychosocial mechanisms driving CDC among Chinese nurses by empirically validating a chain mediation model grounded in Social Cognitive Theory. We demonstrate that EI not only directly enhances CDC (*β* = 0.38, *p* < 0.001) but also exerts a significant indirect effect through the sequential pathway of CSE and SDL, collectively accounting for 23.16% of the total effect. Critically, this model reveals how intrapersonal resources translate into clinical judgment—a cornerstone of patient safety in complex healthcare environments.

### 4.1 EI as the catalyst: from emotional awareness to decisional efficiency

Our finding of a moderate positive correlation between EI and CDC (*r* = 0.491, *p* < 0.001) aligns with SCT’ s core premise that self-regulatory capabilities shape performance outcomes. Crucially, EI transcends mere correlation by enabling nurses to decode emotional cues during high-stakes decisions (e.g., recognizing subtle signs of patient deterioration amid family distress), thereby reducing cognitive load and mitigating decision paralysis—a phenomenon empirically linked to diagnostic errors (29, 30). While our effect size exceeds some Western cohorts (*r* = 0.38) (31), this likely reflects contextual factors unique to Chinese healthcare, such as collectivist cultural values prioritizing emotional harmony. This underscores EI’ s role as a foundational competency for clinical judgment—not merely a correlate. Consequently, nursing administrators must integrate EI development into core curricula through: (1) Simulation-based EI training using standardized scenarios with embedded emotional triggers (e.g., managing distressed families during resuscitation), augmented by real-time biofeedback to regulate physiological stress responses (32). (2) Structured reflective practice where nurses analyze emotional patterns in clinical decisions through peer-facilitated debriefing, normalizing emotional experiences without compromising clinical objectivity (33).

### 4.2 CSE and SDL: the psychological bridge to competence

The 23.16% total indirect effect via the EI → CSE → SDL → CDC pathway highlights CSE’ s pivotal role as a psychological catalyst. SCT posits that EI strengthens CSE by enabling nurses to reframe clinical uncertainties as solvable challenges (e.g., interpreting patient non-adherence as an opportunity for culturally tailored care co-design rather than personal failure). However, CSE alone is insufficient without SDL: Nurses with high CSE actively engage in SDL (e.g., seeking evidence-based strategies to refine clinical judgments), which consolidates decision-making skills through deliberate practice (34, 35). This explains why EI without CSE/SDL may heighten anxiety in ambiguous situations (36)—emotional awareness alone cannot compensate for deficient problem-solving pathways. Our data challenge the assumption that technical competence alone drives CDC. While prior studies emphasize knowledge acquisition (37), CSE and SDL collectively account for significant CDC variance independent of clinical experience (Table 3). This necessitates a paradigm shift in nursing education: (1) Embedding innovation micro-credentials into orientation programs, where nurses earn recognition for implementing evidence-based process improvements (e.g., redesigning handoff protocols); (2) Structured error analysis forums focused on non-critical decision errors, fostering vicarious learning to build CSE without compromising psychological safety (38).

### 4.3 SDL: the critical link between EI and decision-making confidence

Although SDL’ s association with clinical reasoning is established, few studies position it as a direct predictor of clinical decision-making confidence in nursing. This study bridges that gap by demonstrating that SDL significantly mediates the EI → CDC relationship, with CSE serving as its critical antecedent (16, 17). SDL—defined as proactive goal-setting, strategic knowledge acquisition, and reflective evaluation—strengthens CDC by enabling nurses to systematically refine clinical judgment through autonomous learning (39). This mechanism is deeply rooted in SCT: EI fosters CSE (e.g., belief in innovative problem-solving), which in turn motivates SDL engagement. Nurses with higher CSE actively seek learning opportunities to address knowledge gaps, thereby consolidating expertise and confidence. Notably, our findings extend Zhoc et al. work (14) by contextualizing this pathway within acute-care nursing. While Zhoc identified EI as a predictor of SDL in educational settings, we demonstrate its clinical relevance: EI enhances SDL by facilitating anxiety regulation during complex learning tasks (e.g., interpreting critical lab values under time pressure), sustaining motivation, and improving self-assessment accuracy. Consequently, SDL cultivates professional autonomy—where nurses perceive greater control over competence development—directly reinforcing CDC. This resolves a key paradox in prior literature: Nurses with high technical skill but low SDL engagement often report confidence deficits due to perceived knowledge stagnation (40). These findings necessitate three evidence-based shifts in practice: (1) Integrate EI and CSE training into curricula (e.g., simulations for emotion regulation during emergencies); (2) Embed metacognitive SDL tools (e.g., AI-driven reflection prompts for clinical cases); (3) Measure SDL outcomes via validated scales (e.g., Nursing SDL Inventory), not self-reported hours. Future research must: (1) Validate the EI → CSE → SDL → CDC pathway across diverse settings (e.g., rural vs. urban ICUs); (2) Explore moderators like workload intensity; (3) Investigate whether SDL interventions directly enhance CDC beyond technical training.

### 4.4 CSE as the foundational mediator: deepening the EI-CDC pathway

CSE serves as the critical foundational mediator that transforms emotional intelligence into actionable clinical decision-making confidence. Unlike traditional cognitive models that view decision-making as a linear analytical process, our findings reveal that CSE operates as the pivotal psychological bridge where emotional competencies are translated into innovative problem-solving capacities. This aligns with Bandura’s social cognitive theory, which posits that self-efficacy beliefs regulate human functioning through cognitive, motivational, affective, and selection processes (41). It operates through two key mechanisms: (1) emotionally intelligent nurses possess enhanced capacity to recognize and regulate their emotional responses during high-stakes clinical situations. This emotional regulation creates cognitive space for innovative thinking, which directly strengthens their belief in generating novel solutions to complex patient care challenges (42). (2) In high-pressure clinical environments, emotionally intelligent nurses with strong CSE transform anxiety into productive energy rather than allowing it to impair judgment. This phenomenon, termed “positive stress reframing” by Sinclair et al. (43). This challenges the traditional hard-soft skills dichotomy, underscoring that CSE-focused interventions in nursing education—rather than isolated technical or emotional training—yield superior outcomes by unifying EI, creativity, and decision-making assurance. Crucially, intervention designs should explicitly foster “positive stress reframing” and flow experiences to convert anxiety into productive energy during high-stakes decisions. Future protocols should embed CSE metrics as core outcomes, moving beyond isolated EI or technical skill training. Randomized controlled trials testing CSE—focused curricula against traditional approaches will determine optimal strategies for cultivating the EI → CSE → CDC pathway in nursing education and professional development.

### 4.5 Contextual moderators of emotional intelligence pathways

The significant moderation effects of department type and years of service underscore the dynamic interplay between contextual factors and psychological mechanisms in shaping clinical decision-making confidence (44). Specifically, the amplified indirect effect of emotional intelligence through creative self-efficacy in high-stress units (e.g., ICU/emergency) reflects how acute, fast-paced environments necessitate innovative problem-solving to manage uncertainty—a phenomenon empirically validated in critical care settings where EI directly fuels adaptive creativity under time pressure (45), whereas the stronger sequential mediation via self-directed learning in non-high-stress departments (e.g., pediatrics/gynecology) highlights the value of reflective, patient-centered learning in settings prioritizing holistic communication, as demonstrated in longitudinal studies of relational leadership fostering autonomous learning in supportive clinical environments (46). Similarly, novice nurses (≤5 years) rely predominantly on the chain mediation pathway—indicating that psychological resources like creative self-efficacy and self-directed learning compensate for limited experience, consistent with evidence that new graduates leverage these resources to bridge theory-practice gaps (47)—while experienced nurses (> 5 years) exhibit a heightened direct effect, suggesting that accumulated clinical knowledge gradually internalizes decision-making processes, reducing dependence on mediated psychological pathways, as observed in skill-acquisition trajectories where expertise automates judgment. These findings challenge one-size-fits-all training approaches and call for context-sensitive interventions, such as stress-adapted creativity workshops for high-pressure units and structured self-directed learning modules for early-career nurses, to optimize decision-making confidence across diverse nursing roles and career stages.

## 5 Limitations and future directions

This study, while providing valuable insights into the psychological mechanisms underlying CDC, has several methodological constraints. First, while the cross-sectional design precludes causal conclusions, the hypothesized chain mediation pathway is theoretically grounded in Social Cognitive Theory and statistically validated through SEM. Future longitudinal studies tracking EI and CDMC over time are recommended to confirm causal mechanisms. Second, the convenience sampling of nurses exclusively from urban tertiary hospitals in Zhejiang province limits generalizability, particularly to rural settings or healthcare systems with divergent cultural contexts (e.g., individualist vs. collectivist societies). The near-exclusive female sample (96.6%) also limits generalizability, warranting investigation in diverse gender contexts. Future research should explore how unit leadership or patient acuity thresholds interact with these pathways. Third, although Harman’ s single-factor test indicated acceptable common method bias (34.71% < 40% threshold), future work must incorporate objective performance metrics (e.g., simulation-based clinical decision accuracy, patient safety indicators) to triangulate self-report findings. Crucially, given that CSE accounts for 48.3% of SDL variance (as evidenced in our model), intervention studies should explicitly target CSE enhancement through structured programs integrating emotional intelligence training with creative problem-solving simulations.

## 6 Conclusion

Nurses’ EI enhanced CDC primarily via sequential mediation through CSE and SDL, accounting for 72.8% of EI’ s total effect. This pathway varied significantly by departmental stress and career stage. In high-stress units (ICU/emergency), CSE mediated EI → CDC more strongly (*β* = 0.28) than in non-high-stress units (*β* = 0.21; pediatrics/gynecology). Conversely, sequential mediation (EI → CSE → SDL → CDC) dominated in non-high-stress settings (*β* = 0.27 vs. 0.20). Career stage further moderated effects: novices (≤5 years) relied predominantly on full sequential mediation (*β* = 0.31), while experienced nurses (> 5 years) showed a strengthened direct EI → CDC effect (*β* = 0.19). Thus, EI’ s contribution to CDC operated dynamically—context-dependent mediation strengths and structural shifts (full sequential vs. direct effects) emerged across stress environments and career trajectories.

## Data Availability

The raw data supporting the conclusions of this article will be made available by the authors, without undue reservation.

## Footnotes

1
https://www.wjx.cn/
